# Guiding histological assessment of uterine lesions using 3D in vitro ultrasonography and stereotaxis

**Published:** 2017-06

**Authors:** Lieselore Vandermeulen, Ann Cornelis, Christina Kjaergaard Rasmussen, Dirk Timmerman, Thierry Van den Bosch

**Affiliations:** Department of Obstetrics and Gynaecology, University Hospitals KU Leuven, 3000 Leuven, Belgium; Department of Obstetrics and Gynaecology, Regional Hospital RZTienen, 3300 Tienen, Belgium; Department of Pathology, Regional, Hospital RZTienen, 3300 Tienen, Belgium; Department of Obstetrics and Gynaecology, Aarhus University Hospital, 8000 Aarhus, Denmark

**Keywords:** adenomyosis, myoma, iGIS, in vitro, in vitro gel instillation sonography, 3D ultrasonography, stereotaxis

## Abstract

**Objective:**

To compare ultrasonographic features of uterine lesions with the findings at macroscopy and microscopy.

**Methods:**

Case series of ten consecutive women undergoing a hysterectomy for uterine pathology. A preoperative transvaginal ultrasound examination was performed. After hysterectomy, the uterus was re-evaluated by 3D in vitro ultrasonography and in vitro gel instillation sonography (iGIS). The lesion of interest was pinpointed by inserting an intramuscular injection needle using a free-hand 2D-ultrasound guided technique to focus the macroscopic and the microscopic examination by the pathologist.

**Results:**

Adenomyosis, benign fibroids and infiltrating endometrial cancer were diagnosed in six, five and one patient, respectively. We found that iGIS improved image quality of in vitro ultrasound. There was a good correlation between the reported ultrasound features and the final histological diagnosis. Some lesions had been misinterpreted during preoperative ultrasonography or at macroscopical examination: e.g. dense myometrial vessels reported as small myometrial cysts at grey scale ultrasound examination; absence of macroscopical lesions in a case of diffuse adenomyosis.

**Conclusions:**

In vitro 3D ultrasonography and iGIS allow for accurate mapping of uterine lesions so that ultrasound features can be matched with final histology. Our series demonstrates some pitfalls in the interpretation of sonographic and macroscopic features of uterine lesions. Stereotaxis of focal uterine lesions could focus histological assessment and reduces examination time for the pathologist.

## Introduction

Myometrial lesions include adenomyosis, fibroids and sarcomas. Adenomyosis is a common gynaecological disorder characterized by the presence of heterotopic endometrial glands and stroma in the myometrium with adjacent smooth muscle hyperplasia. The exact incidence of adenomyosis is unknown. Based on hysterectomy pathology reports the prevalence ranges from 5 to 70%. The discrepancy in prevalence can be attributed to the various diagnostic classifications, different tissue sample sizes and possible pathologist bias (Garcia et al., 2011; Azziz et al., 1989; Bergholt et al., 2001; Bird et al., 1972). The presenting symptoms of adenomyosis are non-specific, ranging from pelvic pain to dysmenorrhea or menorrhagia and may be associated with subfertility and endometriosis (Kissler et al., 2008; Leyendecker et al., 2009). However, many women with adenomyosis remain asymptomatic (Fernandez et al., 2007). Studies have demonstrated that the sensitivity and specificity of transvaginal ultrasound in diagnosing adenomyosis are comparable to those of both MRI and histology (Dueholm, 2006; Van den Bosch et al., 2016).

Diagnostic criteria for adenomyosis include a globular uterus, myometrial asymmetry, myometrial cysts, echogenic lines and buds, hyperechogenic islands, fan-shaped shadowing and an irregular or interrupted endometrial-myometrial junction (Van den Bosch et al., 2015b). Uterine fibroids are the commonest benign uterine tumours, with an estimated incidence of 20%–40% in women during their reproductive years (Ryan et al., 2005; Wallach et al., 2004). Fibroids can be classified into discrete types 0 to 8, according to the FIGO leiomyoma classification system (Munro et al., 2011).

The precise correlation between three-dimensional (3D) ultrasound findings of myometrial lesions and histopathological findings has been sparsely described. Two-dimensional (2D) ultrasound guided stereotaxis has been used in comparing ultrasonography and histology of the junctional zone (Tetlow et al., 1999). Histologic correlation with ultrasound targeted biopsies of the uterus, using pre-operative three-dimensional ultrasound, but without ultrasound guided stereotaxis, has been reported in a study by Luciano et al. (2013). Because at pathological examination only a limited number of slices from the hysterectomy specimen are taken, the lesion may be missed by the pathologists.

Bird et al. (1972) showed that if the pathologist performed an examination with three sections of the uterus, the incidence of adenomyosis was 31 against 61% if six sections were performed.

The combined use of vaginal ultrasonography and histology has been shown to optimize diagnostic accuracy for intracavitary lesions (Van den Bosch et al., 1995; Van den Bosch et al., 2015a). Likewise, we intended to assess the value of combining ultrasonography and histology in the diagnosis of myometrial lesions.

In this article, we describe a new method to optimize the histological diagnosis of adenomyosis and fibroids using 3D in vitro sonography and in vitro gel instillation sonography (iGIS) stereotaxis.

## Methods

In this case series, we included ten consecutive women with US diagnosis of uterine pathology, already scheduled for hysterectomy for gynaecological pathology by one of the gynaecologists at the Department of Obstetrics and Gynaecology of the Regional Hospital RZ Tienen in Belgium from November 2014 to April 2015. Approval of the local Committee of Medical Ethics was obtained before study initiation. Patient informed consent was not required.

The preoperative transvaginal ultrasound examination was performed using a Voluson S8 with a 4-9 MHz transvaginal probe. A 3D volume was acquired and the image in the coronal plane was reconstructed with volume contrast imaging (VCI) set at 2 mm and tomographic ultrasound imaging (TUI) (Votino et al., 2015). For image colour, we used soft sepia, cool blue or grey scale, depending on the best visualization of the lesions.

**Figure 1:**
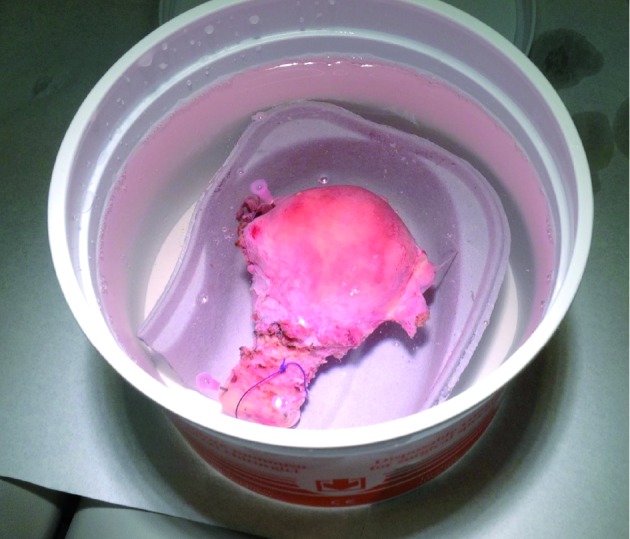
— In vitro setting: the uterus is fixed to a cardboard reniform container and immersed in a 3-liter container filled with water

Immediately after hysterectomy, the uterus was fixed to a cardboard reniform container and immersed in a 3-liter container filled with water (Fig. 1).

The uterus was re-evaluated by the same gynaecologist by in vitro sonography and in vitro gel instillation sonography (iGIS) using a Voluson S8 with a transabdominal 4MHz probe covered with an examination glove. For iGIS a 2.0 mm neonatal suction catheter was inserted through the cervix, after which 3-5 cc Instillagel (® Farco- Pharma GmbH, Köln, Germany) was instilled under ultrasound guidance. To improve orientation and comparison with the histological examination, the lesion of interest was pinpointed by inserting an intramuscular injection needle using a free-hand 2D-ultrasound guided technique (Van den Bosch et al., 2016). Due to his echogenicity, the needle can easily be followed by ultrasonography. The needle is inserted slowly until the tip of the needle is visible in or near the target lesion. The specimen was sent to the pathology laboratory where the uterus was examined at the exact point of the inserted needle by one pathologist. The pathologist was not blinded to the findings of ultrasound examination.

We compared the ultrasound findings before and after the hysterectomy to show the ability to visualize the same myometrial lesions in vivo and in vitro. We also compared the transversal macroscopic sections with the corresponding ultrasound sections and the ultrasound findings with the microscopic results.

## Results

A total of ten patients (six premenopausal, four postmenopausal) were enrolled in this study.

Indications for surgery included menorrhagia or abnormal pre- or postmenopausal bleeding in eight patients, dysmenorrhea/dyspareunia in three patients, and uterine prolapse in one patient. All uteri were re-evaluated by in vitro sonography, five of them also with iGIS. Adenomyosis, benign fibroids and endometrial cancer were diagnosed in six, five patients and one patient, respectively. An overview of the symptoms, ultrasound findings, macroscopic and microscopic examination is presented in Table I.

**Table I:** — Overview of the 10 consecutive cases

	Symptoms	Ultrasonography	Macroscopy	Microscopy	Conclusion/remarks
Case 1	Postmenopausal - uterine prolaps - cystocoele	Myometrial cyst with acoustic enhancement	Cystic lesion suggestive for adenomyosis	Adenomyosis focus with dilated cystic gland	Myometrial cyst at ultrasound examination corresponds to microscopic findings of a cystic dilated cyst. Adenomyosis is more extensive than could have been expected at macroscopic or ultrasound examination.
Case 2	Premenopausal - menorrhagia - ferriprive anaemia	Submucosal FIGO I fibroid in fundus	Submucosal fibroid	Benign submucosal leiomyoma	Perfect correlation between ultrasound, macroscopic and microscopic examination of the large fibroid. Small myometrial ‘cyst’ at ultrasound examination corresponds to a small fibroid on macroscopic and microscopic examination, not with adenomyosis.
		Myometrial 'cyst'	Small fibroid	Small benign leiomyoma	
Case 3	Premenopausal - dysmenorrhea - deep dyspareunia	Myometrial asymmetry: thicker more echogenic posterior wall with numerous small myometrial cysts Irregular and interrupted JZ	Irregular small lesion	Blood vessels	The numerous small myometrial cysts corresponds with blood vessels; - Importance of integration of scale ultrasound imaging and color/power Doppler imaging
		Myometrial cyst	Small cyst	Adenomyosis	
Case 4	Postmenopausal - postmenopausal bleeding	Intramural FIGO 4 fibroid JZ could not be measured	Intramural fibroid	Benign intramural leiomyoma	Lesions, suggestive of adenomyosis on ultrasound and macroscopic examination, correspond to an in ltrating endometrial cancer; - The present of myometrial invasion largely corresponds to the expansion of the endometrial tumour process in adenomyosis
		Echogenic foci	No lesion visible	Well differentiated endometroid adenocarcinoma	
		Nil	Suggestive for adenomyosis	Well differentiated endometroid adenocarcinoma	
		Nil	Large necrotic lesion	Well differentiated endometroid adenocarcinoma, subtype cell variant	
Case 5	Premenopausal - dysmenorrhea - menorrhagia	Echogenic spot	Small nodular lesion	Cystic dilated endometrial gland	Optimal imaging with iGIS. Adenomyosis is more extensive than could have been expected at macroscopic or ultrasound examination.
		Nil	Extra lesion noticed: suggestive for small fibroid	Adenomyoma	
		Nil	Nil	Extra random biopsy; extensive adenomyosis	
Case 6	Premenopausal - menorrhagia - pressure symptoms in suprapubic region	Intramural FIGO 3 fibroid Hematometrium	Intramural fibroid	Benign leiomyoma	iGIS gives extra information about the relationship between fibroid and cavity.
Case 7	Postmenopausal - postmenopausal bleeding	Submucosal FIGO 2 fibroid	Submucosal fibroid	Benign leiomyoma	The macroscopic red pinpoint lesions, macroscopically suggestive for adenomyosis, correspond on microscopy to blood vessels.
			Extra lesion suggestive for adenomyosis	Blood vessels	
Case 8	Premenopausal - menorrhagia - dysmenorrhea - intermenstrual bleeding	Globally enlarged uterus			The marked lesion on ultrasound, suggestive for adenomyosis corresponds with blood vessels. Better image quality with iGIS.
		Endometrioma	Cyst with chocolate- colored fluid, suggestive for endometrioma	Endometrioma	
		Area of increased vascularity	Extra nodular lesion	Adenomyosis	
Case 9	Premenopausal - menorrhagia	Enlarged uterus myomatosus with an intramural FIGO 4 fibroid	Intramural fibroid	Benign leiomyoma	Optimal imaging with iGIS. The marked myometrial cyst corresponds to adenomyosis. Adenomyosis is more extensive than could have been expected at macroscopic or ultrasound examination.
		Myometrial cyst	Red cystic lesion	Adenomyosis	
		Nil	3 smaller intramural fibroids (not noticed on US)	3 adenomyomas	
Case 10	Postmenopausal - persistent vaginal bleeding and abdominal pain despite hormonal therapy	Myometrial cyst with hyperechogenic rim	Multiple cysts	Adenomyosis	Optimal imaging with iGIS (better visualisation of the cornua). Perfect correlation between ultrasound, macroscopy and microscopy. Notwithstanding the presence of extensive adenomyosis, we notice a regular JZ! Although more images of the junctional zone through TUI and VCI are required to examine the entire JZ: e.g. visualisation of echogenic lines and buds.
		Microcysts	Multiple small microcysts	Adenomyosis	

**Figure 2:**
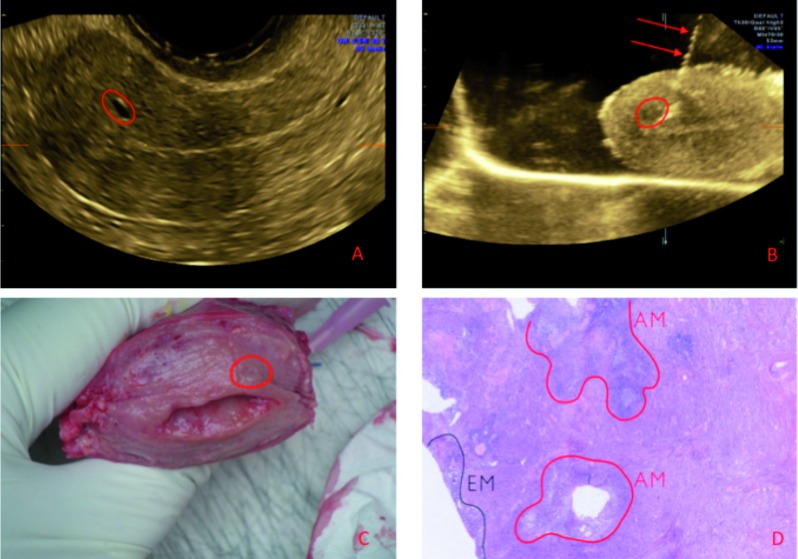
— Case #1: (A) Two-dimensional ultrasound image of the uterus in transverse plane showing a myometrial cyst in the anterior uterine wall. (B) In vitro under water ultrasound examination of the uterus in longitudinal section: the needle (red arrows) is caudal of the myometrial cyst (circle). (C) Macroscopy: in the anterior wall we notice a cyst suggestive for adenomyosis. (D) Microscopy of the needle-marked zone, corresponding to an adenomyosis focus with a dilated cystic gland. EM=endometrium; AM= adenomyosis.

To illustrate the different methods and pathologies, three cases are discussed in more detail:

The first case (case #1) is a 49-year old, postmenopausal woman with a cystocoele and a uterine prolapse. On the pre-operative scan the presence of a myometrial cyst suggested adenomyosis. At macroscopic examination, a cyst in the anterior myometrial wall was confirmed. This was verified at microscopy, though the adenomyosis was more extensive than expected based on ultrasonography or macroscopy. The ultrasound findings and the corresponding macroscopic and microscopic findings are presented in Figure 2. From this case we can conclude that although there was a good correlation between the reported ultrasound features and the final diagnosis on histology of a myometrial adenomyosis cyst, the adenomyosis was more extensive than could have been expected on ultrasound or macroscopy.

**Figure 3:**
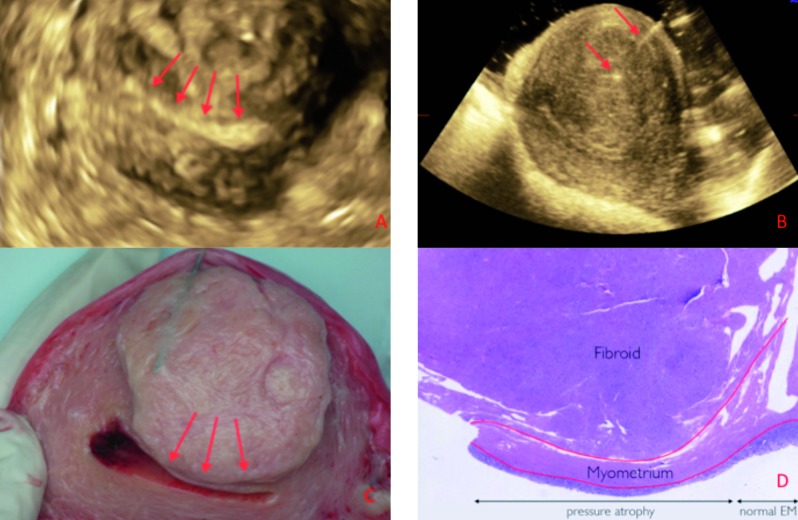
— Case #7: (A) Three-dimensional ultrasound imaging of the uterus in transversal section using VCI, showing the impression of the fibroid on the uterine cavity. (B) In vitro under water ultrasound examination: the needle (arrows) points to the centre of the fibroid (47x45 mm). (C) Transverse section of the hysterectomy specimen (the red arrows indicating the impression of the broid on the endometrium. (D) Microscopy of the submucosal fibroid compressing the myometrium and causing pressure atrophy of the endometrium.

The second case (case #7) is a 63-year old patient presenting with postmenopausal bleeding. Ultrasonography showed a globulous uterus myomatosus with a prominent FIGO type 2 fibroid of 41x40x42 mm in the anterior wall and a thickened endometrium. Histological examination of the endometrial sample showed complex endometrial hyperplasia with atypia. The patient underwent an abdominal hysterectomy. At macroscopy, a large submucosal fibroid in the anterior wall was seen with impression on the uterine cavity/endometrium. The latter was confirmed at microscopy showing pressure atrophy of the endometrium (Fig. 3). From this case we can conclude that there was a good correlation between the reported ultrasound features and the final diagnosis on histology.

The last case (case #10) is a 56-year old postmenopausal woman on cyclic hormonal therapy with persistent vaginal bleeding and dysmenorrhea. On pre-operative scan the diagnosis of adenomyosis was made based on the presence of myometrial cysts with hyperechogenic rim, hypoechogenic microcysts, linear striations and echogenic buds.

**Figure 4:**
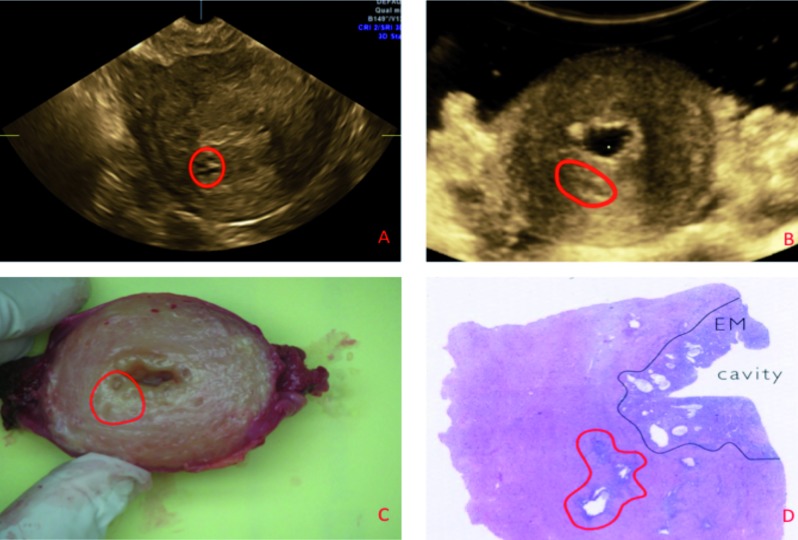
— Case #10: (A) Two-dimensional ultrasound image of the uterus in transverse plane showing myometrial cysts in the posterior wall (red circle). (B) In vitro under water ultrasound examination in transverse plane after gel instillation (iGIS). Notice the presence of the microcysts (red circle). (C) Macroscopy: transverse section of the hysterectomy specimen showing multiple cysts (red circle). (D) Microscopic overview of the on ultrasound marked region. Notice the presence of an adenomyosis cyst (red circle).

On macroscopic examination, we noticed multiple cysts and microcysts, suggestive for adenomyosis. This was confirmed on microscopy (Fig. 4). From this last case we conclude that iGIS enhances image quality and that there was a perfect match between ultrasound, macroscopy and microscopy.

## Discussion

In this study, we illustrated that in vitro ultrasonography with or without iGIS allows for accurate mapping of uterine lesions. We also demonstrated the feasibility of in vitro underwater 3D-ultrasound examination and stereotaxis on a hysterectomy specimen using a free-hand 2D-ultrasound guided technique (Van den Bosch et al., 2016).

This new approach allows to indicate the precise location of the suspected lesions to the pathologist and hence to compare the ultrasound features with macroscopic and microscopic examination.

In this study, in vitro gel instillation sonography was used to optimize image quality and to provide additional information about the lesion and the uterine cavity. To our knowledge this is the first report on iGIS.

For myometrial lesions and especially in adenomyosis ultrasound findings and pathology results are often not concordant. Because only a limited number of slices of the uterus are examined by the pathologist a lesion may be missed at pathology. By using in vitro underwater ultrasound examination of the uterus after hysterectomy and stereotactic needle localization under ultrasound guidance, it is possible to indicate to the pathologist where to expect the (e.g. adenomyosis) lesions.

The best correlation between ultrasound findings and histology was found for fibroids and myometrial cysts. However some (adenomyosis) lesions have been missed on ultrasound. Our series demonstrates possible pitfalls in the interpretation of sonographic and macroscopic features of the uterine lesions. For example, dense myometrial vessels were reported as small myometrial cysts at grey scale ultrasound examination, highlighting the importance of the integration of colour/power Doppler imaging. Furthermore some lesions were also missed at macroscopy in a case of diffuse adenomyosis.

We reported one case of an infiltrating endometrial carcinoma, not diagnosed on the pre-operative scan and with negative sampling. The presence of the myometrial invasion largely corresponded to the expansion of the endometrial tumour process in adenomyosis. Adenocarcinoma arising from adenomyosis is a rare entity and the diagnosis is often difficult. In the literature only a few cases have been described (Taga et al., 2014).

Our study has some limitations. The number of cases is relatively small and the image quality of in vitro ultrasonography could have been improved using a high frequency probe and a high-end ultrasound system. Because the aim of in vitro underwater ultrasound examination and iGIS was to indicate the exact location of the lesion and hence optimize histological assessment, the pathologist was not blinded for the ultrasound findings. To prove the added value of this technique, there is need for prospective randomized trials.

Underwater stereotactic needle insertion under ultrasound guidance is technically simple and can be completed within 10 minutes. The proposed methodology can be used in future studies correlating ultrasound features and histology. Stereotaxis using iGIS may also prove to be useful in guiding the histological assessment of uterine lesions e.g. in oncology. The question whether in vitro underwater stereotaxis may improve diagnostic accuracy or could reduce examination time for the pathologist should be addressed in a larger and prospective study.

## References

[1] Azziz R (1989). Adenomyosis: current perspectives.. Obstet Gynecol Clin North Am.

[2] Bergholt T, Eriksen L, Berendt N (2001). Prevalence and risk factors of adenomyosis at hysterectomy.. Hum reprod.

[3] Bird CC, McElin TW, Manalo-Estrella P (1972). The elusive adenomyosis of the uterus revisited.. Am J Obstet Gynecol.

[4] Dueholm M (2006). Transvaginal ultrasound for diagnosis of adenomyosis: a review.. Best Pract Res Clin Obstet Gynaecol.

[5] Fernandez H, Donnadieu AC (2007). Adenomyosis.. J Gynecol Obstet Biol reprod.

[6] Garcia L, Isaacson K (2011). Adenomyosis: review of the literature.. J Minim Invasive Gynecol.

[7] Kissler S, Zangos S, Hohl J (2008). Duration of dysmenorrhoea and extent of adenomyosis visualised by magnetic resonance imaging.. Eur J Obstet Gynecol Reprod Biol.

[8] Leyendecker G, Wildt L, Mall G (2009). The pathophysiology of endometriosis and adenomyosis: tissue injury and repair.. Arch Gynecol Obstet.

[9] Luciano DE, Exacoustos C, Albrecht L (2013). Three-dimensional ultrasound in diagnosis of adenomyosis: histologic correlation with ultrasound targeted biopsies of the uterus.. J Minim Invasive Gynecol.

[10] Munro MG, Critchley HO, Broder MS; for the FIGO Working Group on Menstrual Disorders FIGO classiffication system (Palm-COeIn) for causes of abnormal uterine bleeding in nongravid women of reproductive age Int J Gynaecol Obstet 2011 113 3 13 21345435 10.1016/j.ijgo.2010.11.011

[11] Ryan GL, Syrop CH, Van Voorhis BJ (2005). Role, epidemiology, and natural history of benign uterine mass lesions.. Clin Obstet Gynecol.

[12] Taga S, Sawada M, Nagai A (2014). A case of endometrioid adenocarcinoma arising from adenomyosis.. Case rep Obstet Gynecol.

[13] Tetlow RL, Richmond I, Manton DJ (1999). Histological analysis of the uterine junctional zone as seen by transvaginal ultrasound.. Ultrasound Obstet Gynecol.

[14] Van den Bosch T, Vandendael A, Van Schoubroeck D (1995). Combining vaginal ultrasonography and office endometrial sampling in the diagnosis of endometrial disease in postmenopausal women. Obstet Gynecol.

[15] Van den Bosch T, Votino A, Cornelis A (2016). Optimizing the Histological diagnosis of adenomyosis using in vitro Three-dimensional ultrasonography.. Gynecol Obstet Invest.

[16] Van den Bosch T, Ameye L, Van Schoubroeck D (2015a). Intra- cavitary uterine pathology in women with abnormal uterine bleeding: a prospective study of 1220 women.. Facts Views Vis Obgyn.

[17] Van den Bosch T, Dueholm M, Leone F (2015b). Terms, definitions and measurements to describe sonographic features of myometrium and uterine masses: a consensus opinion from the morphological uterus Sonographic assessment (muSa) group.. Ultrasound Obstet Gynecol.

[18] Votino A, Van den Bosch T, Installé AJ (2015). Optimizing the ultrasound visualization of the endometrial-myometrial junction (EMJ).. Facts Views Vis Obgyn.

[19] Wallach EE, Vlahos NF (2004). Uterine myomas: an overview of development, clinical features, and management.. Obstet Gynecol.

